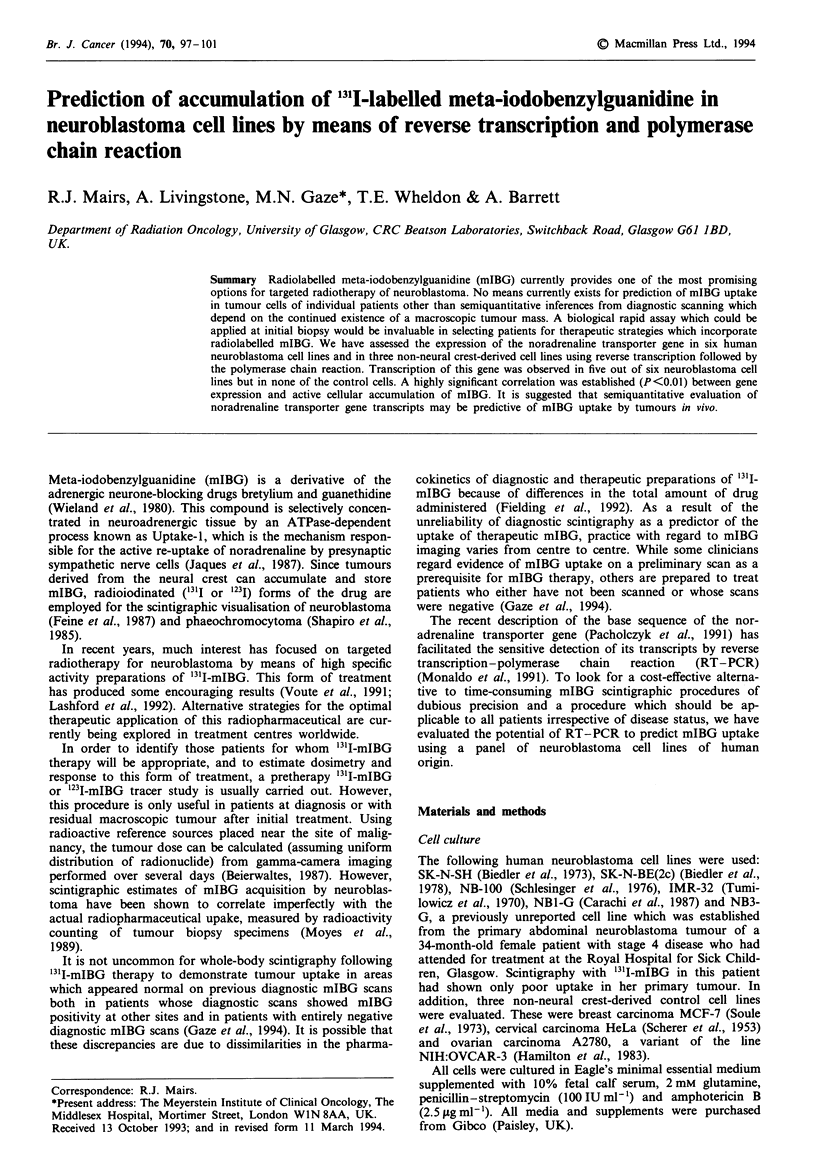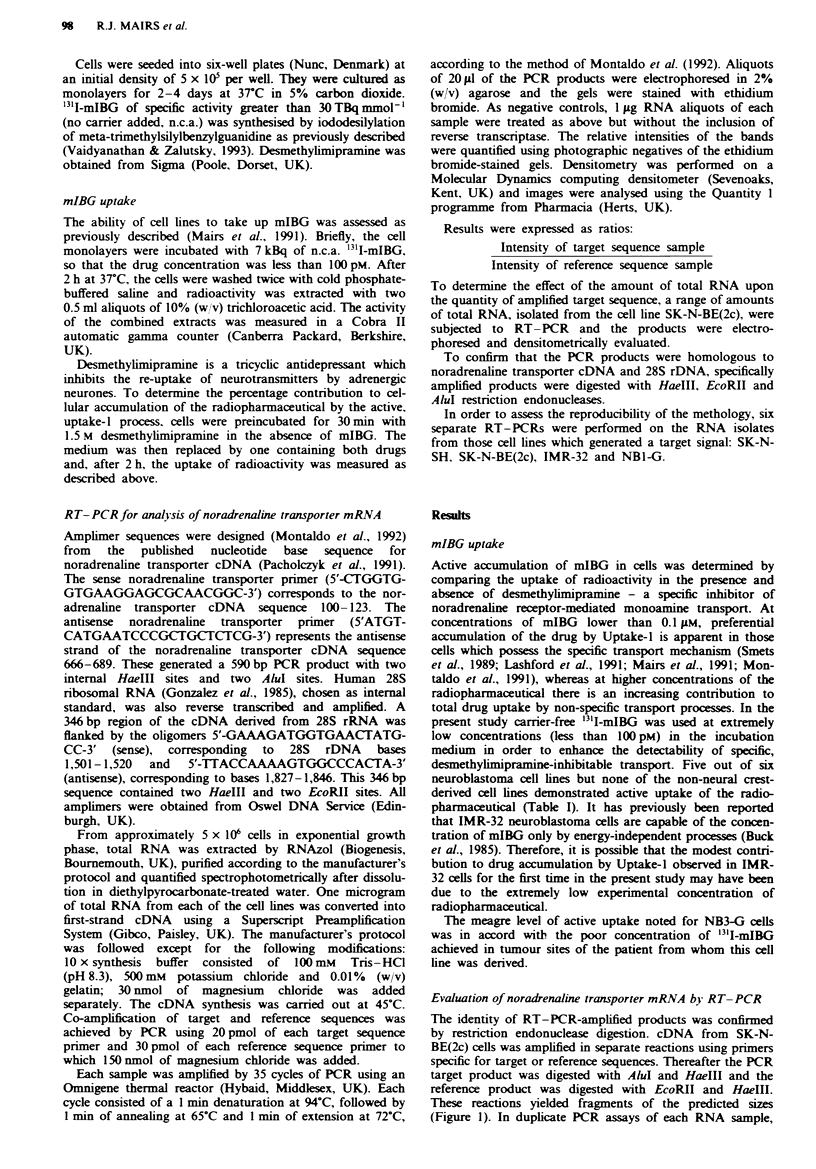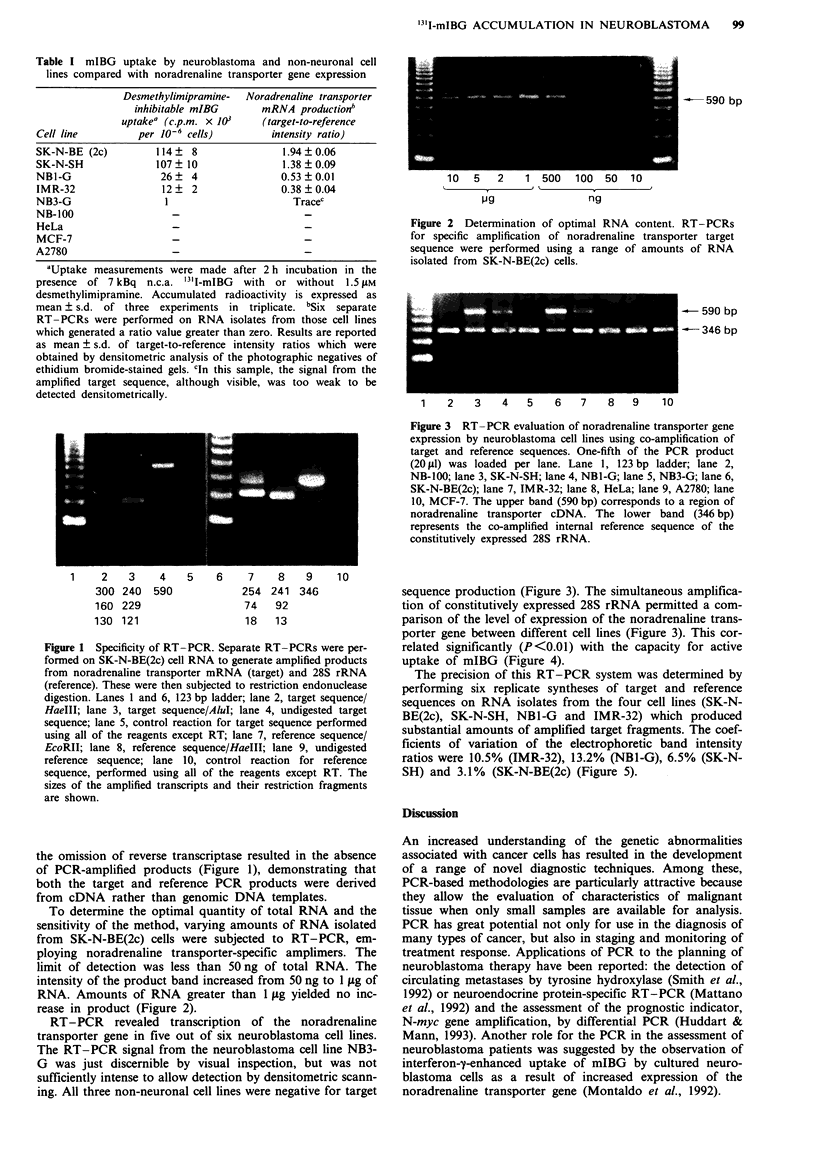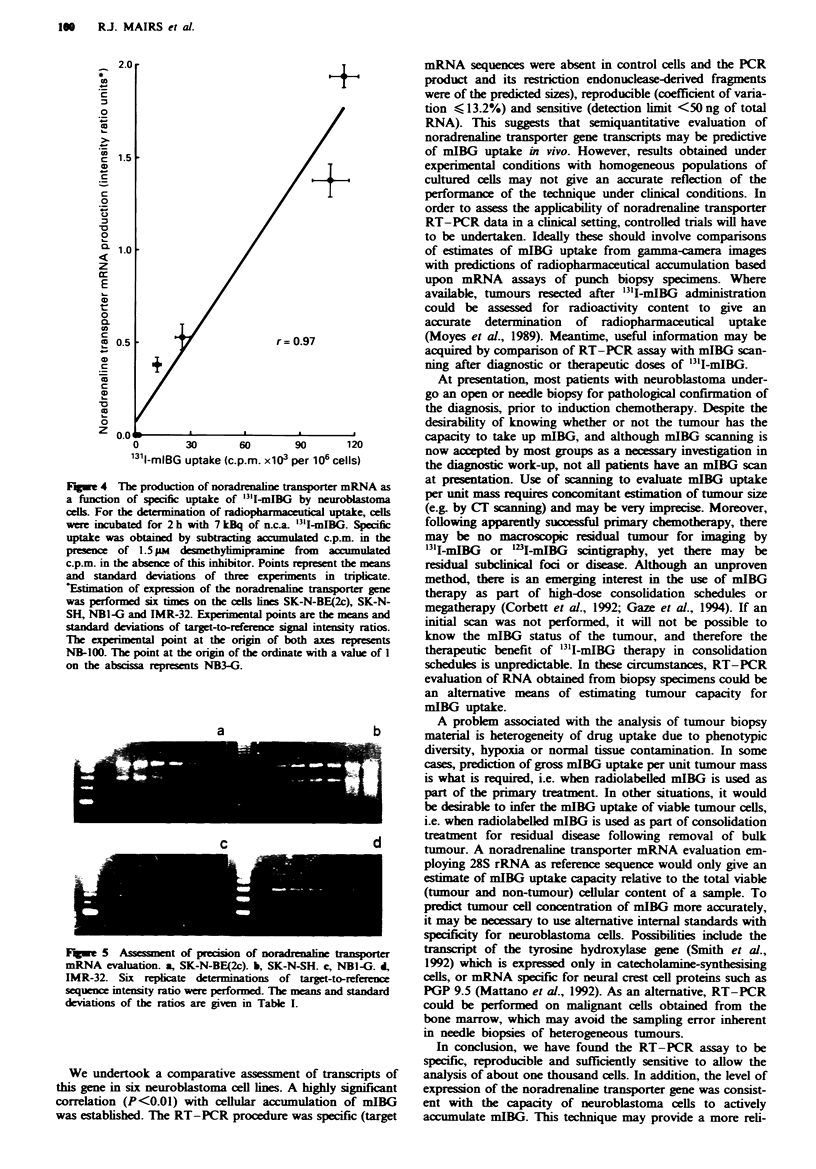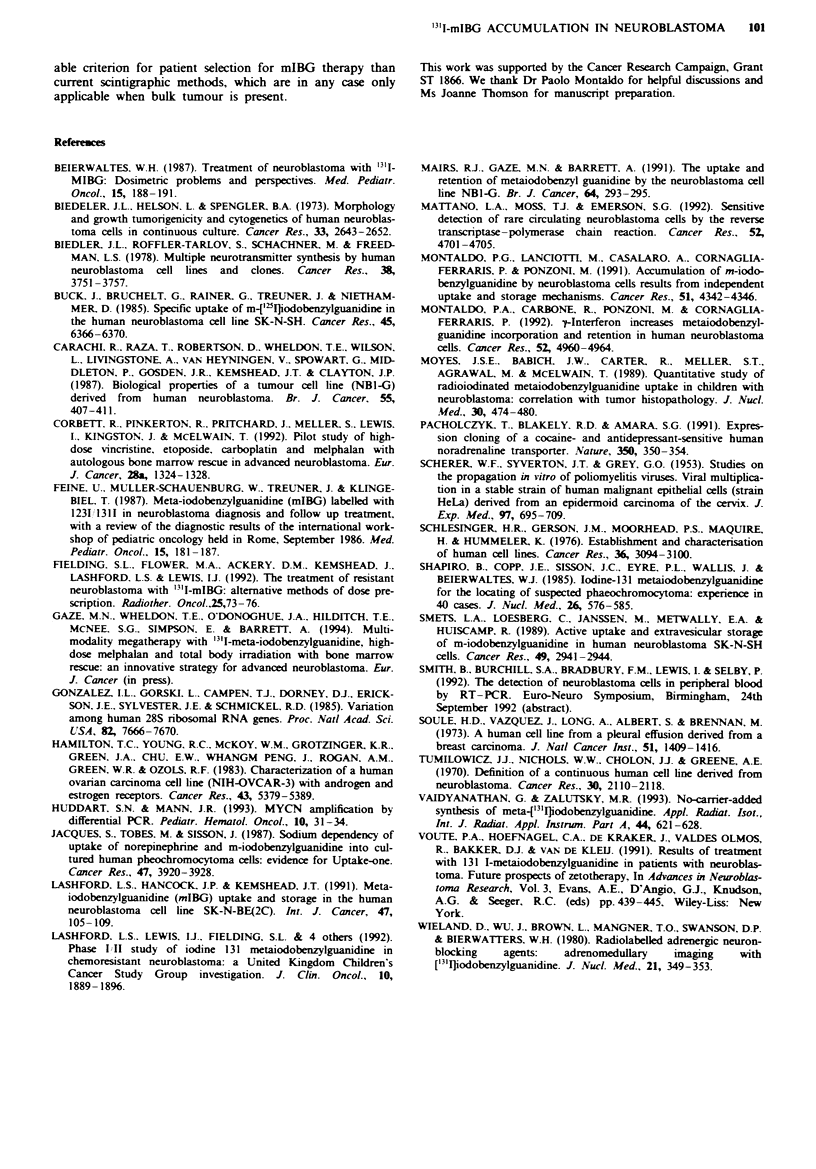# Prediction of accumulation of 131I-labelled meta-iodobenzylguanidine in neuroblastoma cell lines by means of reverse transcription and polymerase chain reaction.

**DOI:** 10.1038/bjc.1994.256

**Published:** 1994-07

**Authors:** R. J. Mairs, A. Livingstone, M. N. Gaze, T. E. Wheldon, A. Barrett

**Affiliations:** Department of Radiation Oncology, University of Glasgow, UK.

## Abstract

**Images:**


Br. J. Cancer (1994), 70, 97-101 © Macmillan Press Ltd., 1994

### Prediction of accumulation of $^{131}$I-labelled meta-iodobenzylguanidine in neuroblastoma cell lines by means of reverse transcription and polymerase chain reaction

R.J. Mairs, A. Livingstone, M.N. Gaze*, T.E. Wheldon & A. Barrett

*Department of Radiation Oncology, University of Glasgow, CRC Beatson Laboratories, Switchback Road, Glasgow G61 1BD, UK.*

**Summary** Radiolabelled meta-iodobenzylguanidine (mIBG) currently provides one of the most promising options for targeted radiotherapy of neuroblastoma. No means currently exists for prediction of mIBG uptake in tumour cells of individual patients other than semiquantitative inferences from diagnostic scanning which depend on the continued existence of a macroscopic tumour mass. A biological rapid assay which could be applied at initial biopsy would be invaluable in selecting patients for therapeutic strategies which incorporate radiolabelled mIBG. We have assessed the expression of the noradrenaline transporter gene in six human neuroblastoma cell lines and in three non-neural crest-derived cell lines using reverse transcription followed by the polymerase chain reaction. Transcription of this gene was observed in five out of six neuroblastoma cell lines but in none of the control cells. A highly significant correlation was established ($P<0.01$) between gene expression and active cellular accumulation of mIBG. It is suggested that semiquantitative evaluation of noradrenaline transporter gene transcripts may be predictive of mIBG uptake by tumours *in vivo*.

Meta-iodobenzylguanidine (mIBG) is a derivative of the adrenergic neurone-blocking drugs bretylium and guanethidine (Wieland *et al*., 1980). This compound is selectively concentrated in neuroadrenergic tissue by an ATPase-dependent process known as Uptake-1, which is the mechanism responsible for the active re-uptake of noradrenaline by presynaptic sympathetic nerve cells (Jaques *et al*., 1987). Since tumours derived from the neural crest can accumulate and store mIBG, radioiodinated ($^{131}$I or $^{123}$I) forms of the drug are employed for the scintigraphic visualisation of neuroblastoma (Feine *et al*., 1987) and phaeochromocytoma (Shapiro *et al*., 1985).

In recent years, much interest has focused on targeted radiotherapy for neuroblastoma by means of high specific activity preparations of $^{131}$I-mIBG. This form of treatment has produced some encouraging results (Voute *et al*., 1991; Lashford *et al*., 1992). Alternative strategies for the optimal therapeutic application of this radiopharmaceutical are currently being explored in treatment centres worldwide.

In order to identify those patients for whom $^{131}$I-mIBG therapy will be appropriate, and to estimate dosimetry and response to this form of treatment, a pretherapy $^{131}$I-mIBG or $^{123}$I-mIBG tracer study is usually carried out. However, this procedure is only useful in patients at diagnosis or with residual macroscopic tumour after initial treatment. Using radioactive reference sources placed near the site of malignancy, the tumour dose can be calculated (assuming uniform distribution of radionuclide) from gamma-camera imaging performed over several days (Beierwaltes, 1987). However, scintigraphic estimates of mIBG acquisition by neuroblastoma have been shown to correlate imperfectly with the actual radiopharmaceutical upake, measured by radioactivity counting of tumour biopsy specimens (Moyes *et al*., 1989).

It is not uncommon for whole-body scintigraphy following $^{131}$I-mIBG therapy to demonstrate tumour uptake in areas which appeared normal on previous diagnostic mIBG scans both in patients whose diagnostic scans showed mIBG positivity at other sites and in patients with entirely negative diagnostic mIBG scans (Gaze *et al*., 1994). It is possible that these discrepancies are due to dissimilarities in the pharmacokinetics of diagnostic and therapeutic preparations of $^{131}$I-mIBG because of differences in the total amount of drug administered (Fielding *et al*., 1992). As a result of the unreliability of diagnostic scintigraphy as a predictor of the uptake of therapeutic mIBG, practice with regard to mIBG imaging varies from centre to centre. While some clinicians regard evidence of mIBG uptake on a preliminary scan as a prerequisite for mIBG therapy, others are prepared to treat patients who either have not been scanned or whose scans were negative (Gaze *et al*., 1994).

The recent description of the base sequence of the noradrenaline transporter gene (Pacholczyk *et al*., 1991) has facilitated the sensitive detection of its transcripts by reverse transcription-polymerase chain reaction (RT-PCR) (Monaldo *et al*., 1991). To look for a cost-effective alternative to time-consuming mIBG scintigraphic procedures of dubious precision and a procedure which should be applicable to all patients irrespective of disease status, we have evaluated the potential of RT-PCR to predict mIBG uptake using a panel of neuroblastoma cell lines of human origin.

### Materials and methods

### *Cell culture*

The following human neuroblastoma cell lines were used: SK-N-SH (Biedler *et al*., 1973), SK-N-BE(2c) (Biedler *et al*., 1978), NB-100 (Schlesinger *et al*., 1976), IMR-32 (Tumilowicz *et al*., 1970), NB1-G (Carachi *et al*., 1987) and NB3-G, a previously unreported cell line which was established from the primary abdominal neuroblastoma tumour of a 34-month-old female patient with stage 4 disease who had attended for treatment at the Royal Hospital for Sick Children, Glasgow. Scintigraphy with $^{131}$I-mIBG in this patient had shown only poor uptake in her primary tumour. In addition, three non-neural crest-derived control cell lines were evaluated. These were breast carcinoma MCF-7 (Soule *et al*., 1973), cervical carcinoma HeLa (Scherer *et al*., 1953) and ovarian carcinoma A2780, a variant of the line NIH:OVCAR-3 (Hamilton *et al*., 1983).

All cells were cultured in Eagle's minimal essential medium supplemented with 10% fetal calf serum, 2 mM glutamine, penicillin-streptomycin (100 IU ml$^{-1}$) and amphotericin B (2.5 μg ml$^{-1}$). All media and supplements were purchased from Gibco (Paisley, UK).

Correspondence: R.J. Mairs.
*Present address: The Meyerstein Institute of Clinical Oncology, The Middlesex Hospital, Mortimer Street, London W1N 8AA, UK.
Received 13 October 1993; and in revised form 11 March 1994.

Cells were seeded into six-well plates (Nunc, Denmark) at an initial density of $5 \times 10^5$ per well. They were cultured as monolayers for 2–4 days at 37°C in 5% carbon dioxide. $^{131}$I-mIBG of specific activity greater than 30 TBq mmol$^{-1}$ (no carrier added, n.c.a.) was synthesised by iododesilylation of meta-trimethylsilylbenzylguanidine as previously described (Vaidyanathan & Zalutsky, 1993). Desmethylimipramine was obtained from Sigma (Poole, Dorset, UK).

### *mIBG uptake*

The ability of cell lines to take up mIBG was assessed as previously described (Mairs *et al.*, 1991). Briefly, the cell monolayers were incubated with 7 kBq of n.c.a. $^{131}$I-mIBG, so that the drug concentration was less than 100 pM. After 2 h at 37°C, the cells were washed twice with cold phosphate-buffered saline and radioactivity was extracted with two 0.5 ml aliquots of 10% (w/v) trichloroacetic acid. The activity of the combined extracts was measured in a Cobra II automatic gamma counter (Canberra Packard, Berkshire, UK).

Desmethylimipramine is a tricyclic antidepressant which inhibits the re-uptake of neurotransmitters by adrenergic neurones. To determine the percentage contribution to cellular accumulation of the radiopharmaceutical by the active, uptake-1 process, cells were preincubated for 30 min with 1.5 M desmethylimipramine in the absence of mIBG. The medium was then replaced by one containing both drugs and, after 2 h, the uptake of radioactivity was measured as described above.

### *RT–PCR for analysis of noradrenaline transporter mRNA*

Amplimer sequences were designed (Montaldo *et al.*, 1992) from the published nucleotide base sequence for noradrenaline transporter cDNA (Pacholczyk *et al.*, 1991). The sense noradrenaline transporter primer (5'-CTGGTG-GTGAAGGAGCGCAACGGC-3') corresponds to the noradrenaline transporter cDNA sequence 100–123. The antisense noradrenaline transporter primer (5'ATGT-CATGAATCCCGCTGCTCTCG-3') represents the antisense strand of the noradrenaline transporter cDNA sequence 666–689. These generated a 590 bp PCR product with two internal *Hae*III sites and two *Alu*I sites. Human 28S ribosomal RNA (Gonzalez *et al.*, 1985), chosen as internal standard, was also reverse transcribed and amplified. A 346 bp region of the cDNA derived from 28S rRNA was flanked by the oligomers 5'-GAAAGATGGTGAACTATG-CC-3' (sense), corresponding to 28S rDNA bases 1,501–1,520 and 5'-TTACCAAAAGTGGCCCACTA-3' (antisense), corresponding to bases 1,827–1,846. This 346 bp sequence contained two *Hae*III and two *Eco*RII sites. All amplimers were obtained from Oswel DNA Service (Edinburgh, UK).

From approximately $5 \times 10^6$ cells in exponential growth phase, total RNA was extracted by RNAzol (Biogenesis, Bournemouth, UK), purified according to the manufacturer's protocol and quantified spectrophotometrically after dissolution in diethylpyrocarbonate-treated water. One microgram of total RNA from each of the cell lines was converted into first-strand cDNA using a Superscript Preamplification System (Gibco, Paisley, UK). The manufacturer's protocol was followed except for the following modifications: 10 × synthesis buffer consisted of 100 mM Tris–HCl (pH 8.3), 500 mM potassium chloride and 0.01% (w/v) gelatin; 30 nmol of magnesium chloride was added separately. The cDNA synthesis was carried out at 45°C. Co-amplification of target and reference sequences was achieved by PCR using 20 pmol of each target sequence primer and 30 pmol of each reference sequence primer to which 150 nmol of magnesium chloride was added.

Each sample was amplified by 35 cycles of PCR using an Omnigene thermal reactor (Hybaid, Middlesex, UK). Each cycle consisted of a 1 min denaturation at 94°C, followed by 1 min of annealing at 65°C and 1 min of extension at 72°C, according to the method of Montaldo *et al.* (1992). Aliquots of 20 µl of the PCR products were electrophoresed in 2% (w/v) agarose and the gels were stained with ethidium bromide. As negative controls, 1 µg RNA aliquots of each sample were treated as above but without the inclusion of reverse transcriptase. The relative intensities of the bands were quantified using photographic negatives of the ethidium bromide-stained gels. Densitometry was performed on a Molecular Dynamics computing densitometer (Sevenoaks, Kent, UK) and images were analysed using the Quantity 1 programme from Pharmacia (Herts, UK).

Results were expressed as ratios:

$$\frac{\text{Intensity of target sequence sample}}{\text{Intensity of reference sequence sample}}$$

To determine the effect of the amount of total RNA upon the quantity of amplified target sequence, a range of amounts of total RNA, isolated from the cell line SK-N-BE(2c), were subjected to RT–PCR and the products were electrophoresed and densitometrically evaluated.

To confirm that the PCR products were homologous to noradrenaline transporter cDNA and 28S rDNA, specifically amplified products were digested with *Hae*III, *Eco*RII and *Alu*I restriction endonucleases.

In order to assess the reproducibility of the methology, six separate RT–PCRs were performed on the RNA isolates from those cell lines which generated a target signal: SK-N-SH, SK-N-BE(2c), IMR-32 and NB1-G.

### Results

### *mIBG uptake*

Active accumulation of mIBG in cells was determined by comparing the uptake of radioactivity in the presence and absence of desmethylimipramine – a specific inhibitor of noradrenaline receptor-mediated monoamine transport. At concentrations of mIBG lower than 0.1 µM, preferential accumulation of the drug by Uptake-1 is apparent in those cells which possess the specific transport mechanism (Smets *et al.*, 1989; Lashford *et al.*, 1991; Mairs *et al.*, 1991; Montaldo *et al.*, 1991), whereas at higher concentrations of the radiopharmaceutical there is an increasing contribution to total drug uptake by non-specific transport processes. In the present study carrier-free $^{131}$I-mIBG was used at extremely low concentrations (less than 100 pM) in the incubation medium in order to enhance the detectability of specific, desmethylimipramine-inhibitable transport. Five out of six neuroblastoma cell lines but none of the non-neural crest-derived cell lines demonstrated active uptake of the radiopharmaceutical (Table I). It has previously been reported that IMR-32 neuroblastoma cells are capable of the concentration of mIBG only by energy-independent processes (Buck *et al.*, 1985). Therefore, it is possible that the modest contribution to drug accumulation by Uptake-1 observed in IMR-32 cells for the first time in the present study may have been due to the extremely low experimental concentration of radiopharmaceutical.

The meagre level of active uptake noted for NB3-G cells was in accord with the poor concentration of $^{131}$I-mIBG achieved in tumour sites of the patient from whom this cell line was derived.

### *Evaluation of noradrenaline transporter mRNA by RT–PCR*

The identity of RT–PCR-amplified products was confirmed by restriction endonuclease digestion. cDNA from SK-N-BE(2c) cells was amplified in separate reactions using primers specific for target or reference sequences. Thereafter the PCR target product was digested with *Alu*I and *Hae*III and the reference product was digested with *Eco*RII and *Hae*III. These reactions yielded fragments of the predicted sizes (Figure 1). In duplicate PCR assays of each RNA sample,

**Table I** mIBG uptake by neuroblastoma and non-neuronal cell lines compared with noradrenaline transporter gene expression

| *Cell line* | *Desmethylimipramine-inhibitable mIBG uptake*[a] *(c.p.m.* $\times 10^3$ *per* $10^{-6}$ *cells)* | *Noradrenaline transporter mRNA production*[b] *(target-to-reference intensity ratio)* |
|---|---|---|
| SK-N-BE (2c) | 114 ± 8 | 1.94 ± 0.06 |
| SK-N-SH | 107 ± 10 | 1.38 ± 0.09 |
| NB1-G | 26 ± 4 | 0.53 ± 0.01 |
| IMR-32 | 12 ± 2 | 0.38 ± 0.04 |
| NB3-G | 1 | Trace[c] |
| NB-100 | – | – |
| HeLa | – | – |
| MCF-7 | – | – |
| A2780 | – | – |

[a]Uptake measurements were made after 2 h incubation in the presence of 7 kBq n.c.a. $^{131}$I-mIBG with or without 1.5 μM desmethylimipramine. Accumulated radioactivity is expressed as mean ± s.d. of three experiments in triplicate. [b]Six separate RT–PCRs were performed on RNA isolates from those cell lines which generated a ratio value greater than zero. Results are reported as mean ± s.d. of target-to-reference intensity ratios which were obtained by densitometric analysis of the photographic negatives of ethidium bromide-stained gels. [c]In this sample, the signal from the amplified target sequence, although visible, was too weak to be detected densitometrically.

**Figure 1** Specificity of RT–PCR. Separate RT–PCRs were performed on SK-N-BE(2c) cell RNA to generate amplified products from noradrenaline transporter mRNA (target) and 28S rRNA (reference). These were then subjected to restriction endonuclease digestion. Lanes 1 and 6, 123 bp ladder; lane 2, target sequence/*Hae*III; lane 3, target sequence/*Alu*I; lane 4, undigested target sequence; lane 5, control reaction for target sequence performed using all of the reagents except RT; lane 7, reference sequence/*Eco*RII; lane 8, reference sequence/*Hae*III; lane 9, undigested reference sequence; lane 10, control reaction for reference sequence, performed using all of the reagents except RT. The sizes of the amplified transcripts and their restriction fragments are shown.

the omission of reverse transcriptase resulted in the absence of PCR-amplified products (Figure 1), demonstrating that both the target and reference PCR products were derived from cDNA rather than genomic DNA templates.

To determine the optimal quantity of total RNA and the sensitivity of the method, varying amounts of RNA isolated from SK-N-BE(2c) cells were subjected to RT–PCR, employing noradrenaline transporter-specific amplimers. The limit of detection was less than 50 ng of total RNA. The intensity of the product band increased from 50 ng to 1 μg of RNA. Amounts of RNA greater than 1 μg yielded no increase in product (Figure 2).

RT–PCR revealed transcription of the noradrenaline transporter gene in five out of six neuroblastoma cell lines. The RT–PCR signal from the neuroblastoma cell line NB3-G was just discernible by visual inspection, but was not sufficiently intense to allow detection by densitometric scanning. All three non-neuronal cell lines were negative for target

**Figure 2** Determination of optimal RNA content. RT–PCRs for specific amplification of noradrenaline transporter target sequence were performed using a range of amounts of RNA isolated from SK-N-BE(2c) cells.

**Figure 3** RT–PCR evaluation of noradrenaline transporter gene expression by neuroblastoma cell lines using co-amplification of target and reference sequences. One-fifth of the PCR product (20 μl) was loaded per lane. Lane 1, 123 bp ladder; lane 2, NB-100; lane 3, SK-N-SH; lane 4, NB1-G; lane 5, NB3-G; lane 6, SK-N-BE(2c); lane 7, IMR-32; lane 8, HeLa; lane 9, A2780; lane 10, MCF-7. The upper band (590 bp) corresponds to a region of noradrenaline transporter cDNA. The lower band (346 bp) represents the co-amplified internal reference sequence of the constitutively expressed 28S rRNA.

sequence production (Figure 3). The simultaneous amplification of constitutively expressed 28S rRNA permitted a comparison of the level of expression of the noradrenaline transporter gene between different cell lines (Figure 3). This correlated significantly ($P < 0.01$) with the capacity for active uptake of mIBG (Figure 4).

The precision of this RT–PCR system was determined by performing six replicate syntheses of target and reference sequences on RNA isolates from the four cell lines (SK-N-BE(2c), SK-N-SH, NB1-G and IMR-32) which produced substantial amounts of amplified target fragments. The coefficients of variation of the electrophoretic band intensity ratios were 10.5% (IMR-32), 13.2% (NB1-G), 6.5% (SK-N-SH) and 3.1% (SK-N-BE(2c) (Figure 5).

### Discussion

An increased understanding of the genetic abnormalities associated with cancer cells has resulted in the development of a range of novel diagnostic techniques. Among these, PCR-based methodologies are particularly attractive because they allow the evaluation of characteristics of malignant tissue when only small samples are available for analysis. PCR has great potential not only for use in the diagnosis of many types of cancer, but also in staging and monitoring of treatment response. Applications of PCR to the planning of neuroblastoma therapy have been reported: the detection of circulating metastases by tyrosine hydroxylase (Smith *et al.*, 1992) or neuroendocrine protein-specific RT–PCR (Mattano *et al.*, 1992) and the assessment of the prognostic indicator, N-*myc* gene amplification, by differential PCR (Huddart & Mann, 1993). Another role for the PCR in the assessment of neuroblastoma patients was suggested by the observation of interferon-γ-enhanced uptake of mIBG by cultured neuroblastoma cells as a result of increased expression of the noradrenaline transporter gene (Montaldo *et al.*, 1992).

**Figure 4** The production of noradrenaline transporter mRNA as a function of specific uptake of $^{131}$I-mIBG by neuroblastoma cells. For the determination of radiopharmaceutical uptake, cells were incubated for 2 h with 7 kBq of n.c.a. $^{131}$I-mIBG. Specific uptake was obtained by subtracting accumulated c.p.m. in the presence of 1.5 $\mu$M desmethylimipramine from accumulated c.p.m. in the absence of this inhibitor. Points represent the means and standard deviations of three experiments in triplicate. *Estimation of expression of the noradrenaline transporter gene was performed six times on the cells lines SK-N-BE(2c), SK-N-SH, NB1-G and IMR-32. Experimental points are the means and standard deviations of target-to-reference signal intensity ratios. The experimental point at the origin of both axes represents NB-100. The point at the origin of the ordinate with a value of 1 on the abscissa represents NB3-G.

**Figure 5** Assessment of precision of noradrenaline transporter mRNA evaluation. **a**, SK-N-BE(2c). **b**, SK-N-SH. **c**, NB1-G. **d**, IMR-32. Six replicate determinations of target-to-reference sequence intensity ratio were performed. The means and standard deviations of the ratios are given in Table I.

We undertook a comparative assessment of transcripts of this gene in six neuroblastoma cell lines. A highly significant correlation ($P<0.01$) with cellular accumulation of mIBG was established. The RT-PCR procedure was specific (target mRNA sequences were absent in control cells and the PCR product and its restriction endonuclease-derived fragments were of the predicted sizes), reproducible (coefficient of variation $\leqslant 13.2\%$) and sensitive (detection limit <50 ng of total RNA). This suggests that semiquantitative evaluation of noradrenaline transporter gene transcripts may be predictive of mIBG uptake *in vivo*. However, results obtained under experimental conditions with homogeneous populations of cultured cells may not give an accurate reflection of the performance of the technique under clinical conditions. In order to assess the applicability of noradrenaline transporter RT-PCR data in a clinical setting, controlled trials will have to be undertaken. Ideally these should involve comparisons of estimates of mIBG uptake from gamma-camera images with predictions of radiopharmaceutical accumulation based upon mRNA assays of punch biopsy specimens. Where available, tumours resected after $^{131}$I-mIBG administration could be assessed for radioactivity content to give an accurate determination of radiopharmaceutical uptake (Moyes *et al*., 1989). Meantime, useful information may be acquired by comparison of RT-PCR assay with mIBG scanning after diagnostic or therapeutic doses of $^{131}$I-mIBG.

At presentation, most patients with neuroblastoma undergo an open or needle biopsy for pathological confirmation of the diagnosis, prior to induction chemotherapy. Despite the desirability of knowing whether or not the tumour has the capacity to take up mIBG, and although mIBG scanning is now accepted by most groups as a necessary investigation in the diagnostic work-up, not all patients have an mIBG scan at presentation. Use of scanning to evaluate mIBG uptake per unit mass requires concomitant estimation of tumour size (e.g. by CT scanning) and may be very imprecise. Moreover, following apparently successful primary chemotherapy, there may be no macroscopic residual tumour for imaging by $^{131}$I-mIBG or $^{123}$I-mIBG scintigraphy, yet there may be residual subclinical foci or disease. Although an unproven method, there is an emerging interest in the use of mIBG therapy as part of high-dose consolidation schedules or megatherapy (Corbett *et al*., 1992; Gaze *et al*., 1994). If an initial scan was not performed, it will not be possible to know the mIBG status of the tumour, and therefore the therapeutic benefit of $^{131}$I-mIBG therapy in consolidation schedules is unpredictable. In these circumstances, RT-PCR evaluation of RNA obtained from biopsy specimens could be an alternative means of estimating tumour capacity for mIBG uptake.

A problem associated with the analysis of tumour biopsy material is heterogeneity of drug uptake due to phenotypic diversity, hypoxia or normal tissue contamination. In some cases, prediction of gross mIBG uptake per unit tumour mass is what is required, i.e. when radiolabelled mIBG is used as part of the primary treatment. In other situations, it would be desirable to infer the mIBG uptake of viable tumour cells, i.e. when radiolabelled mIBG is used as part of consolidation treatment for residual disease following removal of bulk tumour. A noradrenaline transporter mRNA evaluation employing 28S rRNA as reference sequence would only give an estimate of mIBG uptake capacity relative to the total viable (tumour and non-tumour) cellular content of a sample. To predict tumour cell concentration of mIBG more accurately, it may be necessary to use alternative internal standards with specificity for neuroblastoma cells. Possibilities include the transcript of the tyrosine hydroxylase gene (Smith *et al*., 1992) which is expressed only in catecholamine-synthesising cells, or mRNA specific for neural crest cell proteins such as PGP 9.5 (Mattano *et al*., 1992). As an alternative, RT-PCR could be performed on malignant cells obtained from the bone marrow, which may avoid the sampling error inherent in needle biopsies of heterogeneous tumours.

In conclusion, we have found the RT-PCR assay to be specific, reproducible and sufficiently sensitive to allow the analysis of about one thousand cells. In addition, the level of expression of the noradrenaline transporter gene was consistent with the capacity of neuroblastoma cells to actively accumulate mIBG. This technique may provide a more reliable criterion for patient selection for mIBG therapy than current scintigraphic methods, which are in any case only applicable when bulk tumour is present.

This work was supported by the Cancer Research Campaign, Grant ST 1866. We thank Dr Paolo Montaldo for helpful discussions and Ms Joanne Thomson for manuscript preparation.

### References

BEIERWALTES, W.H. (1987). Treatment of neuroblastoma with $^{131}$I-MIBG: Dosimetric problems and perspectives. *Med. Pediatr. Oncol.*, **15**, 188–191.

BIEDELER, J.L., HELSON, L. & SPENGLER, B.A. (1973). Morphology and growth tumorigenicity and cytogenetics of human neuroblastoma cells in continuous culture. *Cancer Res.*, **33**, 2643–2652.

BIEDLER, J.L., ROFFLER-TARLOV, S., SCHACHNER, M. & FREEDMAN, L.S. (1978). Multiple neurotransmitter synthesis by human neuroblastoma cell lines and clones. *Cancer Res.*, **38**, 3751–3757.

BUCK, J., BRUCHELT, G., RAINER, G., TREUNER, J. & NIETHAMMER, D. (1985). Specific uptake of m-[$^{125}$I]iodobenzylguanidine in the human neuroblastoma cell line SK-N-SH. *Cancer Res.*, **45**, 6366–6370.

CARACHI, R., RAZA, T., ROBERTSON, D., WHELDON, T.E., WILSON, L., LIVINGSTONE, A., VAN HEYNINGEN, V., SPOWART, G., MIDDLETON, P., GOSDEN, J.R., KEMSHEAD, J.T. & CLAYTON, J.P. (1987). Biological properties of a tumour cell line (NB1-G) derived from human neuroblastoma. *Br. J. Cancer*, **55**, 407–411.

CORBETT, R., PINKERTON, R., PRITCHARD, J., MELLER, S., LEWIS, I., KINGSTON, J. & MCELWAIN, T. (1992). Pilot study of high-dose vincristine, etoposide, carboplatin and melphalan with autologous bone marrow rescue in advanced neuroblastoma. *Eur. J. Cancer*, **28a**, 1324–1328.

FEINE, U., MULLER-SCHAUENBURG, W., TREUNER, J. & KLINGEBIEL, T. (1987). Meta-iodobenzylguanidine (mIBG) labelled with 123I/131I in neuroblastoma diagnosis and follow up treatment, with a review of the diagnostic results of the international workshop of pediatric oncology held in Rome, September 1986. *Med. Pediatr. Oncol.*, **15**, 181–187.

FIELDING, S.L., FLOWER, M.A., ACKERY, D.M., KEMSHEAD, J., LASHFORD, L.S. & LEWIS, I.J. (1992). The treatment of resistant neuroblastoma with $^{131}$I-mIBG: alternative methods of dose prescription. *Radiother. Oncol.*,**25**,73–76.

GAZE, M.N., WHELDON, T.E., O'DONOGHUE, J.A., HILDITCH, T.E., MCNEE, S.G., SIMPSON, E. & BARRETT, A. (1994). Multimodality megatherapy with $^{131}$I-meta-iodobenzylguanidine, high-dose melphalan and total body irradiation with bone marrow rescue: an innovative strategy for advanced neuroblastoma. *Eur. J. Cancer* (in press).

GONZALEZ, I.L., GORSKI, L., CAMPEN, T.J., DORNEY, D.J., ERICKSON, J.E., SYLVESTER, J.E. & SCHMICKEL, R.D. (1985). Variation among human 28S ribosomal RNA genes. *Proc. Natl Acad. Sci. USA*, **82**, 7666–7670.

HAMILTON, T.C., YOUNG, R.C., MCKOY, W.M., GROTZINGER, K.R., GREEN, J.A., CHU, E.W., WHANGM PENG, J., ROGAN, A.M., GREEN, W.R. & OZOLS, R.F. (1983). Characterization of a human ovarian carcinoma cell line (NIH-OVCAR-3) with androgen and estrogen receptors. *Cancer Res.*, **43**, 5379–5389.

HUDDART, S.N. & MANN, J.R. (1993). MYCN amplification by differential PCR. *Pediatr. Hematol. Oncol.*, **10**, 31–34.

JACQUES, S., TOBES, M. & SISSON, J. (1987). Sodium dependency of uptake of norepinephrine and m-iodobenzylguanidine into cultured human pheochromocytoma cells: evidence for Uptake-one. *Cancer Res.*, **47**, 3920–3928.

LASHFORD, L.S., HANCOCK, J.P. & KEMSHEAD, J.T. (1991). Meta-iodobenzylguanidine (*m*IBG) uptake and storage in the human neuroblastoma cell line SK-N-BE(2C). *Int. J. Cancer*, **47**, 105–109.

LASHFORD, L.S., LEWIS, I.J., FIELDING, S.L. & 4 others (1992). Phase I/II study of iodine 131 metaiodobenzylguanidine in chemoresistant neuroblastoma: a United Kingdom Children's Cancer Study Group investigation. *J. Clin. Oncol.*, **10**, 1889–1896.

MAIRS, R.J., GAZE, M.N. & BARRETT, A. (1991). The uptake and retention of metaiodobenzyl guanidine by the neuroblastoma cell line NB1-G. *Br. J. Cancer*, **64**, 293–295.

MATTANO, L.A., MOSS, T.J. & EMERSON, S.G. (1992). Sensitive detection of rare circulating neuroblastoma cells by the reverse transcriptase-polymerase chain reaction. *Cancer Res.*, **52**, 4701–4705.

MONTALDO, P.G., LANCIOTTI, M., CASALARO, A., CORNAGLIA-FERRARIS, P. & PONZONI, M. (1991). Accumulation of *m*-iodobenzylguanidine by neuroblastoma cells results from independent uptake and storage mechanisms. *Cancer Res.*, **51**, 4342–4346.

MONTALDO, P.A., CARBONE, R., PONZONI, M. & CORNAGLIA-FERRARIS, P. (1992). γ-Interferon increases metaiodobenzylguanidine incorporation and retention in human neuroblastoma cells. *Cancer Res.*, **52**, 4960–4964.

MOYES, J.S.E., BABICH, J.W., CARTER, R., MELLER, S.T., AGRAWAL, M. & MCELWAIN, T. (1989). Quantitative study of radioiodinated metaiodobenzylguanidine uptake in children with neuroblastoma: correlation with tumor histopathology. *J. Nucl. Med.*, **30**, 474–480.

PACHOLCZYK, T., BLAKELY, R.D. & AMARA, S.G. (1991). Expression cloning of a cocaine- and antidepressant-sensitive human noradrenaline transporter. *Nature*, **350**, 350–354.

SCHERER, W.F., SYVERTON, J.T. & GREY, G.O. (1953). Studies on the propagation *in vitro* of poliomyelitis viruses. Viral multiplication in a stable strain of human malignant epithelial cells (strain HeLa) derived from an epidermoid carcinoma of the cervix. *J. Exp. Med.*, **97**, 695–709.

SCHLESINGER, H.R., GERSON, J.M., MOORHEAD, P.S., MAQUIRE, H. & HUMMELER, K. (1976). Establishment and characterisation of human cell lines. *Cancer Res.*, **36**, 3094–3100.

SHAPIRO, B., COPP, J.E., SISSON, J.C., EYRE, P.L., WALLIS, J. & BEIERWALTES, W.J. (1985). Iodine-131 metaiodobenzylguanidine for the locating of suspected phaeochromocytoma: experience in 40 cases. *J. Nucl. Med.*, **26**, 576–585.

SMETS, L.A., LOESBERG, C., JANSSEN, M., METWALLY, E.A. & HUISCAMP, R. (1989). Active uptake and extravesicular storage of m-iodobenzylguanidine in human neuroblastoma SK-N-SH cells. *Cancer Res.*, **49**, 2941–2944.

SMITH, B., BURCHILL, S.A., BRADBURY, F.M., LEWIS, I. & SELBY, P. (1992). The detection of neuroblastoma cells in peripheral blood by RT-PCR. Euro-Neuro Symposium, Birmingham, 24th September 1992 (abstract).

SOULE, H.D., VAZQUEZ, J., LONG, A., ALBERT, S. & BRENNAN, M. (1973). A human cell line from a pleural effusion derived from a breast carcinoma. *J. Natl Cancer Inst.*, **51**, 1409–1416.

TUMILOWICZ, J.J., NICHOLS, W.W., CHOLON, J.J. & GREENE, A.E. (1970). Definition of a continuous human cell line derived from neuroblastoma. *Cancer Res.*, **30**, 2110–2118.

VAIDYANATHAN, G. & ZALUTSKY, M.R. (1993). No-carrier-added synthesis of meta-[$^{131}$I]iodobenzylguanidine. *Appl. Radiat. Isot., Int. J. Radiat. Appl. Instrum. Part A*, **44**, 621–628.

VOUTE, P.A., HOEFNAGEL, C.A., DE KRAKER, J., VALDES OLMOS, R., BAKKER, D.J. & VAN DE KLEIJ. (1991). Results of treatment with 131 I-metaiodobenzylguanidine in patients with neuroblastoma. Future prospects of zetotherapy, In *Advances in Neuroblastoma Research*, Vol. 3, Evans, A.E., D'Angio, G.J., Knudson, A.G. & Seeger, R.C. (eds) pp. 439–445, Wiley-Liss: New York.

WIELAND, D., WU, J., BROWN, L., MANGNER, T.O., SWANSON, D.P. & BIERWATTERS, W.H. (1980). Radiolabelled adrenergic neuron-blocking agents: adrenomedullary imaging with [$^{131}$I]iodobenzylguanidine. *J. Nucl. Med.*, **21**, 349–353.